# First report of the mitochondrial DNA sequences of Chukchi Charr (*Salvelinus andriashevi*, Salmonidae)

**DOI:** 10.1080/23802359.2017.1339211

**Published:** 2017-06-20

**Authors:** Alla G. Oleinik, Lubov A. Skurikhina, Andrey D. Kukhlevsky, Evgeniia I. Bondar

**Affiliations:** a A.V. Zhirmunsky Institute of Marine Biology, National Scientific Center of Marine Biology FEB RAS, Vladivostok, Russia;; b Department of Genetics, Far Eastern Federal University, Vladivostok, Russia

**Keywords:** Salvelinus, Chukchi charr *S. andriashevi*, Taranetz charr *S. taranetzi*, mtDNA genealogy

## Abstract

Three mitochondrial DNA regions were sequenced in two individuals of the Chukchi charr *Salvelinus andriashevi* from Lake Istihed (Chukotka): the entire gene sequences of cytochrome *b* and cytochrome *c* oxidase-1, and control region. The low level of sequence divergence detected between the sequences suggests that *S. andriashevi* is probably an isolated population of the Taranetz charr *S. taranetzi.* The genealogy of mtDNA haplotypes confirms the phylogenetic closeness of *S. andriashevi, S. taranetzi, Salvelinus* sp. 4 (Lake Nachikinskoe), *S. krogiusae* (Lake Dal’nee), *S. boganidae* and *S. elgyticus* (Lake Elgygytgyn), and *S. a. erythrinus* (NWT).

The divergence of phenotypically different and often geographically isolated forms is a significant problem for the taxonomy and phylogeny of charr of the genus *Salvelinus*. A theme that should be considered separately is isolated lacustrine charr populations known in many water bodies of Northeast Asia. Among such charrs is the Chukchi charr, an endemic narrow-range species represented by a single population in land-locked Lake Istihed (64°29' N/173°33' W) in the Chukotka Peninsula (Chereshnev et al. 2002). Originally described as a separate species, *Salvelinus andriashevi* Berg (1948) was subsequently synonymized with *Salvelinus alpinus malma* var. *andriashevi* (Barsukov 1958) or regarded as a subspecies *Salvelinus alpinus* (Glubokovsky et al. 1979). Relying on the identity of karyotypes (Frolov & Frolova 2001) and 22 protein nuclear loci (Omel’chenko et al. 1998) from Istihed and Achchen lakes, it was concluded that the Chukchi charr represents an isolated local population of the Taranetz charr. However, the species status of Chukchi charr was later confirmed based on morphological characters (Chereshnev et al. 2002).

We have sequenced the three mitochondrial DNA (mtDNA) regions, among them those in *S. andriashevi* for the first time, for comparison with other lake charrs and for more precise phylogenetic analysis. Sequences of mtDNA were investigated in 70 specimens of charrs: *S. andriashevi* (Lake Istihed), *S. taranetzi* (Achchen and Pekulineiskoe Lakes, Chukotka), *Salvelinus* sp. 4 (Lake Nachikinskoe, Kamchatka), *S. krogiusae* (Lake Dal’nee, Kamchatka), *S. boganidae* and *S. elgyticus* (Lake Elgygytgyn, Chukotka), *S. alpinus alpinus* (Lake Sitasjaure, Scandinavian), *S. alpinus oquassa* (Floods Pond, North America), *S. alpinus erythrinus* (Jayko and Lauchlan Rivers, Victoria Island, Nunavut, Canada), *S. malma malma* (Paratunka and Kamchatka Rivers; Nachikinskoe, Achchen, and Pekulineiskoe Lakes). The fish specimens are stored in the collection of the Laboratory of Genetics (National Scientific Center of Marine Biology FEB RAS; www.imb.dvo.ru). Following the previous genetic studies with reference to *Salvelinus* species (Crête-Lafrenière et al. 2012; Yamamoto et al. 2014), the entire cytochrome *b* gene (*Cytb*; 1141 bp), a segment of the 5' end of the cytochrome *c* oxidase I gene (*COI*; 1200 bp) and control region (*CR*; 1021 bp) were sequenced using following primers (Ward et al. 2005; Uiblein et al. 2001). All nucleotide sequence data in this study are available from Genbank/NCBI under accession numbers KY1222045-KY122332. Two haplotypes were retrieved from Genbank to add to the analysis (accession numbers: *S. malma malma* KJ746618, *S. alpinus* AF154851).

The sequences are very similar in size and gene arrangement and composition to the charr genomes published previously (Doiron et al. 2002; Balakirev et al. 2016). The total length of mtDNA nucleotide sequences was 3362 bp. The overall base composition was 26.5% A, 29.8% T, 16.6% G and 27.2% C. The combined sequence (1-1021 bp *СR,* 1022-2221 bp *COI,* and 2222-3362 bp *Cytb*) had 124 variable sites (92 parsimony-informative) and 37 different haplotypes. The analyzed mtDNA region in two speciments of *S. andriashevi* (accession numbers: KY122239/KY122045/KY122142 and KY122240/KY122046/KY122143) had one haplotype. The haplotype of *S. andriashevi* is placed in a phylogenetic group combining haplotypes of *S. taranetzi, Salvelinus* sp. 4*, S. krogiusae, S. elgyticus, S. boganidae* and *S. a. erythrinus* (Figure 1). The genealogical distance between this group and haplotypes of *S. m. malma-S. alpinus* was 42 nucleotide substitutions. *S. andriashevi* was most close to *S. taranetzi* from the type locality (Lake Achchen), the differences between the nearest haplotypes were two substitutions. The level of divergence between *S. andriashevi* and other taxa within the phylogenetic group was relatively low (D_xy_ = 0.001 ± 0.000 – 0.003 ± 0.001). These values were somewhat lower than those obtained based on the mtDNA PCR-RFLP analysis but corresponded to the level of intraspecific variability in the genus *Salvelinus* (Oleinik et al. 2015). At the same time, the level of divergence of allopatric *S. andriashevi* and *S. malma malma* (D_xy_ = 0.014 ± 0.002), *S. andriashevi* and *S. alpinus alpinus* (D_xy_ = 0.015 ± 0.002) matched the estimates for allopatric and sympatric populations of *S. taranetzi* and *S. malma malma* (D_xy_ = 0.014 ± 0.002)*, Salvelinus* sp. 4 and *S. malma malma* (D_xy_ = 0.013 ± 0.002)*, S. krogiusae* and *S. malma malma* (D_xy_ = 0.013 ± 0.002). Thus, the ratio of within- to between-population divergence of the mtDNA nucleotide sequences was analogous to that previously reported for *S. taranetzi* and *S. m. malma* populations (Oleinik et al. 2015).

**Figure 1.**
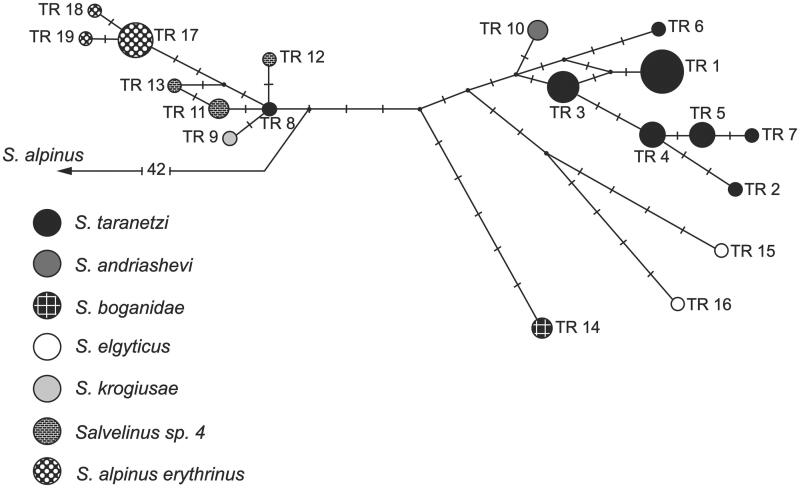
Median-joining network for the charrs of the genus *Salvelinus,* resulting from the analysis of combined sequences of mtDNA (*COI*, *Cytb* genes, and *CR*). Mutational differences between the haplotypes are shown on the branches; the circle size is proportional to the absolute haplotype frequencies. All mutations have equal weights; the interval of the median vector search (black circles), *ɛ* is zero.

Our results suggest that the specimens of Chukchi charr belong to the arctic group of Taranetz charr according to Oleinik et al. (2015). The genealogy of mtDNA haplotypes supports the phylogenetic closeness of *S. andriashevi* with *S. taranetzi* and their recent divergence and/or origin from a common ancestor. *S. andriashevi* is the least diverged in the arctic group and can be regarded as an isolated population of *S. taranetzi.* We encountered the problem of non-conformity of the taxonomic differentiation of charrs based on morphological and genetic analyses. Chereshnev et al. (2002) argued for the independent status of Chukchi charr and emphasized that the degree of the craniological differences between *S. andriashevi* and *S. taranetzi* is comparable to the differences between *S. andriashevi* and *S. malma* and between *S. andriashevi* and *S. alpinus*. The objective reason for the observed discrepancy of morphological and genetic differentiation is likely to be recent divergence of the populations, and/or uneven evolutionary dynamics of qualitatively different characters against the background of exceptional ecological plasticity of charrs (Brunner et al. 2001; Oleinik et al. 2015). Another reason may be limitations imposed by some adaptive morphological characters as phylogenetic markers. Despite the low level of genetic divergence from *S. taranetzi*, the isolated charr population of Lake Istihed is of particular interest for the study of the problem of evolution and speciation in the *Salvelinus* group, as well as for the rational management and biological monitoring of Arctic ecosystems.
